# B-Cell-Mediated Strategies to Fight Chronic Allograft Rejection

**DOI:** 10.3389/fimmu.2013.00444

**Published:** 2013-12-17

**Authors:** Ali Dalloul

**Affiliations:** ^1^INSERM U 996, Clamart, France

**Keywords:** transplantation, chronic rejection, B-cells, regulatory B-cells, immunomodulation

## Abstract

Solid organs have been transplanted for decades. Since the improvement in graft selection and in medical and surgical procedures, the likelihood of graft function after 1 year is now close to 90%. Nonetheless even well-matched recipients continue to need medications for the rest of their lives hence adverse side effects and enhanced morbidity. Understanding Immune rejection mechanisms, is of increasing importance since the greater use of living-unrelated donors and genetically unmatched individuals. Chronic rejection is devoted to T-cells, however the role of B-cells in rejection has been appreciated recently by the observation that B-cell depletion improve graft survival. By contrast however, B-cells can be beneficial to the grafted tissue. This protective effect is secondary to either the secretion of protective antibodies or the induction of B-cells that restrain excessive inflammatory responses, chiefly by local provision of IL-10, or inhibit effector T-cells by direct cellular interactions. As a proof of concept B-cell-mediated infectious transplantation tolerance could be achieved in animal models, and evidence emerged that the presence of such B-cells in transplanted patients correlate with a favorable outcome. Among these populations, regulatory B-cells constitute a recently described population. These cells may develop as a feedback mechanism to prevent uncontrolled reactivity to antigens and inflammatory stimuli. The difficult task for the clinician, is to quantify the respective ratios and functions of “tolerant” vs. effector B-cells within a transplanted organ, at a given time point in order to modulate B-cell-directed therapy. Several receptors at the B-cell membrane as well as signaling molecules, can now be targeted for this purpose. Understanding the temporal expansion of regulatory B-cells in grafted patients and the stimuli that activate them will help in the future to implement specific strategies aimed at fighting chronic allograft rejection.

## Introduction

Kidneys have been the most frequently transplanted organs for decades. Since the improvements in graft selection and in medical and surgical procedures, the likelihood of graft function after 1 year is now close to 90%. Nonetheless even well-matched recipients continue to need medications for the rest of their lives hence adverse side effects and enhanced morbidity. The half-life of transplanted kidneys is still below 10 years indicating a continuous deterioration of the organ due to combined metabolic and immunologic rejection mechanisms. The later are of increasing importance since the greater use of living-unrelated donors and genetically unmatched individuals. Chronic rejection is not specific of kidney transplants but works equally in other grafts and depends on HLA matching. Chronic rejection is mediated by T-cells and often successfully prevented by immunosuppressive drugs aimed at inhibiting T-cell proliferation. However the role of B-cells in chronic rejection has been appreciated recently in patients and in experimental models. Furthermore, the finding that B-cells can also, be beneficial to the grafted tissue generated great interest. This “tolerizing effect” occurs either through the secretion of protective antibodies or through induction of – recently described – regulatory B-cells and secondary induction of regulatory T-cells.

### The role of B-cells in transplant rejection

#### Acute and hyperacute rejection are mediated by preformed or by induced antibodies

##### Induced antibodies

MHC (HLA) antigens and minor antigens that behave like nominal antigens and are presented by HLA molecules to T-cells, are responsible for T-cell alloreactivity. These new determinants expressed on donor tissue ultimately lead to the formation of new antibodies directed at MHC antigens. This T-cell-dependent response generates IgM and then converts to IgG production. These Abs are not responsible for acute graft rejection, however it is now clear that they play a major role in chronic graft destruction (1). They are produced by T-dependent (TD) B2 B-cells with a highly diversified repertoire. This is in contrast with preformed antibodies: anti-HLA Abs occur also following multiple blood transfusions a condition frequently observed in patients on the waiting list for kidney transplantation. Less often they occur as a result of pregnancy or of previous organ transplantation, these Abs are high affinity IgG produced by B2 B-cells. Preformed Antibodies also include natural Abs directed against blood group carbohydrate determinants. These are mostly T-independent (TI) IgM in germline configuration and are produced by a CD5neg B1b B-cell population. Preformed Abs cause hyperacute rejection in vascularized organs such as kidneys and heart. IgM recognize blood groups not only on red cells but also on vascular endothelium, and bind to and activate complement proteins c5–c9, thereby forming the membrane attack complex. This complex activates endothelial cells leading to the loss of antithrombotic molecules and hemorrhage (2) well before cell lysis occurs. Several regulatory molecules such as CD55, CD46, and CD59 control unwanted complement activation (3), however if the affinity of the Abs to the antigen is high enough, it overcomes regulatory molecules of the complement cascade. This is best achieved with IgG anti-HLA Abs. At present there is no treatment of hyperacute rejection, however prevention can be achieved by avoiding blood group disparities, testing the recipient for anti-HLA Abs, so called “cross match” and by plasmapheresis.

#### Chronic rejection

This condition generally takes 5–10 years to develop even in patients treated with immunosuppressive drugs. Several observations have emerged from clinical practice: the process is associated with the presence of anti-donor antibodies and is refractory to increases in immunosuppressive therapy in contrast to acute rejection episodes. There is also a correlation between the onset of chronic rejection and a history of acute rejection episode. Importantly, pathological manifestations include narrowing of vessels associated with proliferation of endothelial cells and smooth muscle cells (4). The media is progressively destroyed, cellular proliferation halts and cells are replaced by concentric fibrosis. In parallel there is increased expression of adhesion molecules and of NOS, aFGF, IGF1, and endothelin. The process is TD and involves cross priming of recipient T-cells with donor peptides presented by recipient antigen presenting cells (APC) (5). However in experimental models alloantibodies in the absence of T-cells can induce the typical vascular changes and lesions can develop in T-cell deficient mice. In other models graft vasculopathy occurs in T-cell-deficient animal allografts but not isografts. Yet, other models imply IFN-gamma-producing T-cells (Th1) and IL-12-unresponsive (Th1 deficient) STAT4^−/−^ animals show markedly reduced severity of graft vasculopathy (6). Furthermore the respective roles of Th1 vs. Th2 responses are far from clear as both populations may be profibrotic and produce TGFbeta. In addition, Th1-produced IFN-gamma has antiproliferative activity and even propapoptotic activity on T-cells.

### B-cells are prominent effectors of allograft rejection

B-cells are positive regulators of the immune response. They differentiate in Ab-producing plasma cells. They are APC and as such can stimulate CD4+ T-cells directly (7) although they fail to stimulate naïve T-cells by contrast with professional (dendritic cells) APC (8). B-cells may also stimulate T-cells by producing cytokines, including IL-4, IL-6, and IFN-gamma. CD4+ T-cell activation is indeed impaired in the absence of B-cells in multiple models where B-cell development is blocked since birth (9, 10). B-cells infiltrate chronically rejected allografts, and several groups have reported the presence of ectopic germinal centers within grafted tissues, so called tertiary lymphoid tissues (11–13). Thus, a likely scenario is that B-cells present antigens to T-cells *in situ* and become activated and proliferate thereby producing a variety of antibodies including anti-self Abs (14). The role of B-cells in chronic graft rejection is not limited to solid organs. In chronic GVHD, B-cells from the donor contribute to the clinical manifestations of the disease and accordingly, B-cell depletion by anti-CD20 MAb/Rituximab improved steroid-resistant chronic GVHD (15). Moreover, responses occurred before the decline in Ab titers again suggesting a cell-mediated effect of B-cells in this process (16). B-cell activation in this setting, is evidenced by elevated BAFF/CD257 levels in patients with active GVHD (17). Although the efficiency of Rituximab is a proof of concept for the role of B-cells in GVHD, the few studies conducted so far have shown limited effect in treating chronic kidney rejection furthermore Rituximab was even deleterious as an induction therapy for acute cellular rejection (18). This suggested that B-cells may be protective in this setting, and strengthened the emerging concept that B-cells within the grafted tissue can be associated with tolerance rather than rejection (19). The “tolerant” or “effector” status of graft-infiltrating B-cells is therefore worth investigating as a prognostic factor in organ transplantation, inasmuch as regulatory B-cells are now much better characterized.

### The concept of “tolerizing” and regulatory- breg- B-cells, against autoimmune diseases may now apply to allogenic transplantation

The observation of immunosuppressive B-cells, able to suppress delayed type hypersensitivity (DTH) dates back to 1974 (20, 21). Since then, B-cells have been shown to inhibit inflammation and autoimmunity in various mouse models. Tolerizing B-cells inhibited effector T-cells whether they have been polarized toward effector Th1, Th17, or Th2 cells (22–24). Their effect was often attributed to local provision of IL-10 although in some instances TGFbeta was the prominent cytokine involved in tolerance induction (25).

The data from this abundant literature were ultimately translated into human studies. A CD19^+^CD24^hi^CD38^hi^IL-10-producing regulatory B-cell subset similar to the murine one was described (26). This subset is functionally impaired in human SLE (27) and possibly in multiple sclerosis (28).

The vast majority of the data implying a protective role for B-cells relates to autoimmune diseases however, increasing results imply B-cells as potential inhibitors of allograft rejection. Indeed a B-cell signature is associated with a positive outcome in renal transplantation patients, and is characterized by increased CD19^+^CD24^hi^CD38^hi^IL-10^+^ B-cells (29). This signature can be useful to detect renal transplant tolerance in humans (30). Thus in given – yet to be determined – conditions, Breg expand in transplanted patients and are able to slow or prevent graft destruction. The other difficulty pertains to the definition of Breg-cells. Although great strides have been made toward understanding their phenotype, IL-10-producing (so called B10) and Breg-cells are not synonymous although these populations clearly overlap.

### Mechanisms of breg induced tolerance/immunosupression

The suppressive capacity of Breg on Th and inflammatory responses has been ascribed chiefly to their secretion of IL-10. This poses the question of how is IL-10 production optimally triggered? In the mouse, B-cells produce high amounts of IL-10 following culture with LPS+ PMA+ Ionomycin, which mimics a combined TLR4 and strong BCR engagement whereas LPS alone induced IL-10 production in plasma cells but not in CD5^+^CD1d^+^ Breg-cells (31). Several other studies demonstrated that BCR-signaling is an important component of IL-10 production. We have observed a direct correlation between the intensity of BCR stimulation and IL-10 secretion by a B-cell line (32). An important outcome of BCR stimulation is a very rapid rise in intracytosolic Ca2+ [c]. Mice with a B-cell restricted deficiency in the stromal interaction molecules STIM-1 and -2 Ca2+ sensors, failed to develop IL-10-producing Breg-cells (33). CD40 is a major effector of Breg activation and IL-10 production, indirectly by inducing the expression of CD5 (32) but also directly as shown by the ability of agonistic CD40 Abs to inhibit the Th1 response via IL-10 production (34). However IL-10 may be dispensable as tolerance induction by anti-CD45RB required B-cells but was only dependent on B-7, CD40, and ICAM-1 expression on B-cells but not of IL-10 production (35). Above all, the main effect of IL-10 may reside in the promotion of regulatory T-cells Treg (36, 37), through induction of Foxp3 (38, 39) although IL-10 may be insufficient in this respect and needs to be associated with cognate CD80/CD86 interactions (40).

The BCR interact with several transmembrane positive (CD19) and negative (CD5 and CD22) regulators of signaling. Among surface inhibitors, FcgRIIb/CD32 is of special interest as a tolerance inducer (41). Its inhibitory activity is chiefly based on its immunotyrosine based inhibitory motif ITIM (42, 43). Its low affinity ensures that it interacts with Immune complexes and not with monomeric ligands (41) thereby explaining the efficacy of aggregated intravenous Ig (IVIG). B-cell stimulation through the BCR can be arrested with colligation (physiologically by immune complexes) of FcgRIIb to the BCR. This results in phosphorylation of ITIM on tyrosine and recruitment of the inositol phosphatase SHIP-1 (44) and arrest of Ca2+ influx (45). Meanwhile the Src kinase Lyn is phosphorylated which results in B-cell tolerance (46). The advantage of CD32 is that it is the only Fc receptor present on B-cells (47), its role in the efficacy of intravenous Ig IVIG to treat idiopathic thrombopenic purpura ITP has been therefore suggested (48). Therefore, co-engagement of the BCR with an inhibitory receptor such as CD32b, CD22 (a molecule with an ITIM domain), or CD5 (a molecule with an atypical ITIM domain) is worth investigating. We have indeed previously shown that BCR cross-linked to an Fc-gammaRIIb-intracytosolic CD5 chimera, in a B-cell line downregulated BCR-mediated early signaling events, notably Ca2+ influx and MAP Kinase activation pathway (49).

### Phenotype of Breg and comparison with other IL-10-producing B-cells

There is now a consensus regarding the phenotype of human Breg [for recent reviews, see (50, 51)]. Breg-cells are CD19^+^IgM^hi^ CD24^h^iCD5^+^CD10^+^CD27^+^CD38^+^CD138^+^CD1d^hi^, and express intracellular IL-10. However they cannot be sorted based on their CD1d^hi^ or CD24^hi^ expression, which does not delineate discrete B-cell subsets (unpublished results). CD10 is a marker of pro/pre B-cells, whereas CD38^+^CD24^hi^ expression marks transitional B-cells (52). Likewise, CD5^+^ B-cells are very heterogeneous and in the adult blood, they are a mixture of transitional B-cells, B1a like B-cells, and activated/memory B2-B-cells (53). In B2-B-cells, CD5 is optimally expressed by stimulation with a combination of CD40-ligand and anti-IgM Abs (32) and produce IL-10. In brief, CD5 is expressed on BCR-engaged B-cells and serves as a negative feed-back to prevent unwanted overreactivity, in part through the secretion of IL-10 and also by downregulating early BCR-signaling. However CD5 expression is transient on B2 B-cells, unless they are triggered permanently by a cognate antigen, such as in the mice double transgenic for Hen Egg Lysozyme HEL and anti-HEL Ig, these animals are tolerant to HEL, however tolerance is broken and autoantibodies produced when the animals are bred into a CD5-null background (54). In contrast, B1a cells which produce natural IgM and IL-10, express “naturally” CD5, however 25% of the recently identified human B1 B-cells do not express surface CD5 (55). Thus, until recently, there were no surface markers exclusive of Breg; the description of T-cell Ig domain and mucin domain TIM-1 on Breg may nevertheless change the picture, inasmuch as TIM-1 ligation induced Breg to promote tolerance in mice (56). The status of the T-cell inhibitory receptor PD-1 in Breg-cells is presently undetermined as it is for PD-Ligands and may be of potential interest since PD-1 is also expressed on B-cells and modulate the humoral immune response [reviewed in Ref. (57)]. Finally, it should be mentioned that although IL-21 is the major cytokine involved in the expansion of B10-cells (58), the level of IL-21R is apparently similar in B10 and non-B10-cells, and thus is by no means a marker for Breg-cells.

### Strategies to inhibit B-cell-mediated rejection

The ultimate goal in transplantation is to achieve tolerance to an allograft; this implies that the transplanted organ remains functional in the absence of immunosuppressive regimen. This is almost never achieved in humans contrary to animal models were adoptively transferred lymphocytes can promote “infectious” tolerance. Current strategies (Table 1) can be summarized in two main objectives: (1) eliminating effector B-cells or inhibiting their differentiation in Ab-producing plasma cells, (2) expanding or stimulating IL-10-producing B-cells/Breg.

**Table 1:** **Potential B-cell targets for the treatment of allograft rejection**.

Target	Ligand	Expected mechanism
CD20	Rituximab, newly developed mAbs	B-cell depletion
B-cell metabolism	Cyclophosphamide, mitoxantrone	B-cell depletion
IL-6	Neutralizing Abs	Inhibition of PC/Ig
Proteasome inhibitors	Bortezomib	Inhibition PC/Ig
JAK3	Pharmacological inhibitors	Inhibition of STATs/IL-6
STAT3	Inhibitors of Y705 phosphorylation	Inhibition of PC differentiation
FcgRIIB	CD32b agonists, IgG2 immune complexes	B-cell tolerance induction
CD22	Agonistic mAbs?	B-cell tolerance induction?
CD5	CD5-BCR cross-linking	Induction of B-cell tolerance, cell cycle arrest?
CD40±BCR	CD40-ligand+anti-sIg	IL-10 production
TLR9	Agonistic mAbs, CpGODN, resiquimod, IL-10 production, B-cell tolerance	
CD25	Interleukin-2	Expansion of CD5+ B-cells, IL-10 production, expansion of Treg cells
BAFF-receptor	Soluble BAFF	Production of IL-10 (B10 B-cells)
TIM-1	Agonistic mAb	Stimulation of Breg-cells

The first task appears difficult owing to the lack of specific target. Currently as stated above, there is no indication that Rituximab can be used to treat chronic graft rejection, however experimental data suggest that the depletion of alloreactive B-cells at the time of the transplantation may promote tolerance by expanding *de novo* B-cells, which become “accustomed” to the grafted organ since the beginning of their development (59). Similarly, a same strategy using cyclophosphamide, mycophenolate mofetil, and mitoxantrone can be implemented in order to achieve a mixed chimerism at the time of transplantation (60, 61). In view of the available data, the value of a B-cell-depleting conditioning regimen at the onset of the transplantation, to ameliorate long term organ acceptance should be considered as an option. In contrast the failure of B-cell depletion strategies once chronic rejection has started implies that redundant immune and inflammatory mechanisms involving various immune cells operate and cannot be halted by B-cell depletion. In this setting, expanding anti-inflammatory/regulatory lymphocytes is currently the best therapeutic option. Yet a strategy to inhibit plasma-cell differentiation in order to damp the production of anti-donor HLA Abs can be envisaged. The proteasome inhibitor bortezomib (62), and neutralizing anti-IL-6 Abs are currently studied as inhibitors of plasma-cell expansion; furthermore the anti-IL-6R MAb, Tocilizumab used in the treatment of Rheumatoid arthritis may be effective in transplantation (63). As STAT3 is a transcription factor needed for plasma-cell differentiation, inhibition of STAT3 (64, 65) may also be of interest. Drugs acting upstream STAT3, namely JAK3 inhibitors seem promising in this respect (66).

It is here worth mentioning that Abs may in some instances be protective. The finding that catalytic antibodies able to disrupt the amplification loop of the coagulation cascade (67) with anti-inflammatory properties (68) may instead pave the way for the selective use of IVIG (69) or immune complexes.

The Inhibitory B-cell signature observed in transplantations with favorable outcome (29, 30) included FcgRIIB (70) suggesting an *in vivo* stimulation of this inhibitory receptor. Another BCR-associated ITIM bearing transmembrane receptor CD22 (71), is a potential B-cell-specific target (72). Breg-cells can expand and be stimulated by CD40 agonists alone or in combination with anti-Ig (34). In addition, whereas cross-linking of CD40 by CD40-ligand induces the maturation of B-cells, prolonged CD40 stimulation inhibits Ig secretion and B-cell differentiation (73). Soluble BAFF was shown to stimulate B10-cells in a mouse model (74) and may be another attractive option. Among inducers of tolerance, the TLR agonists were extensively studied. Agonistic stimulators of TLR-4 or-9 were shown to suppress the course of autoimmune diseases (75–77), which may also explain why infection with salmonella triggered the expansion of IL-10-producing B-cells (78). The well established protective role of apoptotic cells (79) in inducing B10-Breg-cells (80) was highlighted by the observation that MZB and B1 B-cells recognize chromatin complexes and secrete IL-10 through TLR9 transduction pathway (81). As CD5+ B-cells express TLR9, secrete IL-10, and proliferate in response to IL-2 (53), expanding Breg-cells *in vivo* with IL-2 should be considered as a therapeutic option inasmuch as IL-2 also expands CD25+Treg cells (82). It is also inferred from these data that pharmacological inhibitors of NFAT-IL-2 pathway may be detrimental in chronic allograft rejection. The use of Rapamycin an inhibitor of TOR and an inducer of autophagy, instead of cyclosporine and FK506, should therefore be considered (83). Recently, the pivotal role of IL-21 in the expansion of murine B10-cells was demonstrated (58). Interestingly, B10-cells acquire their regulatory functions after they have been activated by IL-21-producing CD4^+^T-cells through CD40L/CD40 and CD4/MHC class-II interactions. This may pave the way for the selection and *ex vivo* expansion of Breg-cells for therapeutic purposes if it turns out that human Breg-cells can be expanded as murine ones.

**Figure 1:**
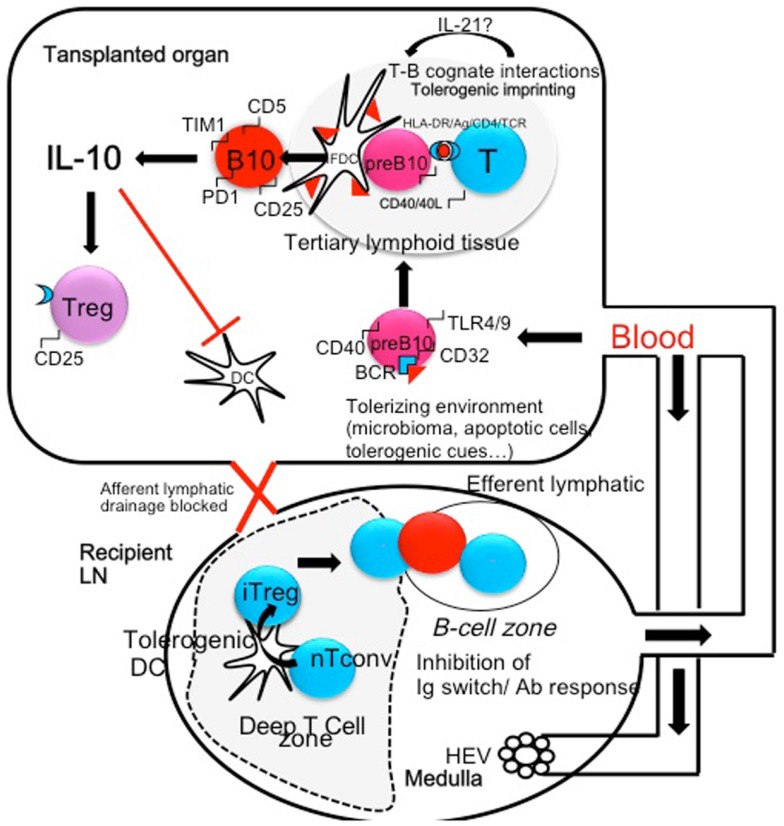
**The microenvironment and/or the microbiome (helminthes, parasites, bacterias) can shift B-cells that recognize microbial antigen in the spleen or in the transplanted organ toward regulatory B-cells (pre B10)**. These cells may be generated in the spleen and colonize the transplanted organ. In turn activated B-cells may present donor MHC peptides to T-cells and undergo cognate T-B interactions in tertiary lymphoid organs through CD40/CD40L, HLA-DR/TCR, and HLA-DR/CD4 interactions. These interactions generate an amplification loop: in the presence of IL-21 secreted by T-cells, B-cells become IL-10-secreting cells, express CD5, TIM1, and PD-1 molecules. In turn, IL-10 inhibits antigen presentation by professional APC (Dendritic cells) and amplifies the production of Treg cells, themselves a potent source of local IL-10. Treg cells migrate to peripheral lymph node through the blood stream and concur to the inhibition of Th1 and Th2 polarization. In addition Treg migrate to the B-cell follicles where they inhibit Antibody secretion and Immunoglobulin isotype switching.

Altogether, experimental data strongly suggest that some immunological conditions such as infections with bacterias or helminths may surprisingly improve tolerance to allografts by repressing harmful lymphocytes and expanding/activating protective cells among which, Breg-cells play a prominent role (Figure 1). As transplanted organs are connected *de novo* to the recipient blood stream but not lymphatics, is it likely that the tolerizing signals are brought to the transplanted tissue through the blood. Tolerant lymphocytes may also traffic, and induce tolerance into peripheral lymph nodes (Figure 1). A better knowledge of the grafted tissue microenvironment and a better appreciation of the local balance between inflammatory and regulatory cells and their temporal fate will undoubtedly lead to improvements in the treatment of chronic allograft rejection. Ultimately *in situ* immunomodulation may tip the balance of intra-graft B-cells toward Breg-cells and possibly expand them locally. Alternatively *ex vivo* expansion followed by injection of Breg-cells within the transplanted organ can be performed. These strategies may become therapeutic options in the future in the case of resistance to immunosuppressive drugs.

### Potential adverse side effects of B-cell-based therapies: Infections

What would be the safety of such therapies? One might expect lesser drawbacks with B-cell-mediated therapies than with pharmacological immunosuppressive regimens especially those pertaining to drug toxicity. However there is no reason to believe that infectious complications similar to those induced by immune-suppression regimen (84) will be avoided. Indeed, activation of Treg cells secondary to that of Breg, may reactivate latent viral infections such as VZV, CMV, EBV, and polyoma (85). Treg cells were also shown to inhibit HCV-specific CD8+ T-cells (86). As mentioned above, infections and the microbiome control many aspects of alloimmunity in transplantation (87). Interestingly, B10 B-cells with a typical Breg phenotype were induced in an experimental model by infecting mice with the red blood cell parasite *Babesia microti* (88); this led to an enhanced susceptibility to infections and associated with a rise in Treg cells, and was not observed in B-cell-deficient mice. Altogether, it is likely that *in vivo* or *ex vivo* amplification of Breg-cells for therapy may lead to enhanced susceptibility to parasitic and possibly bacterial infections.

The role of Interleukin-10 in infections is complex, it may exacerbate T-cell dysfunctions and microbial infections. Indeed, increased circulating IL-10 concentrations have been associated with an adverse clinical outcome in patients with sepsis (89). In contrast some data are consistent with a protective role for IL-10 in kidney (90) lung (91) and heart transplant recipients (92). IL-10 has been shown to protect from septic shock in staphylococcal enterotoxin-induced shock (93). In an experimental model of peritonitis, intravenous injection of Mesenchymal Stem Cells into mice, albeit at a high concentration of 10^6^cells/g body weight, protected animals by triggering IL-10 secretion by macrophages (94). Protection depended only on IL-10 but not of cellular interactions as MSC were trapped into the lungs and did not cross the blood barrier. IL-10 was also shown to control the onset of irreversible septic shock in mice (95). Altogether, strategies aimed at inducing IL-10 secretion by resident intra-graft lymphocytes should be promising and may obviate the adverse effects of systemic IL-10.

## Conclusion

Recent progress in understanding the phenotype and functions of tolerant B- and T-lymphocytes rendered possible their analysis in biopsies of allograft tissues; Based on the discovery of new molecular targets in lymphocytes, implementing new strategies based on redirecting graft-infiltrating lymphocytes toward tolerance in order to fight chronic allograft rejection is becoming foreseeable.

## Conflict of Interest Statement

The author declares that the research was conducted in the absence of any commercial or financial relationships that could be construed as a potential conflict of interest.
